# Integrated Deep Learning and Supervised Machine Learning Model for Predictive Fetal Monitoring

**DOI:** 10.3390/diagnostics12112843

**Published:** 2022-11-17

**Authors:** Vinayaka Gude, Steven Corns

**Affiliations:** 1 Department of Marketing and Business Analytics, Texas A&M University—Commerce, Commerce, TX 75428, USA; 2 Department of Engineering Management and Systems Engineering, Missouri University of Science and Technology, Rolla, MO 65409, USA

**Keywords:** cardiotocography, acidosis, support vector machines, random forests, machine learning, oversampling

## Abstract

Asphyxiation associated with metabolic acidosis is one of the common causes of fetal deaths. The paper aims to develop a feature extraction and prediction algorithm capable of identifying most of the features in the SISPORTO software package and late and variable decelerations. The resulting features were used for classification based on umbilical cord pH data. The algorithms developed here were used to predict cord pH levels. The prediction system assists the obstetricians in assessing the state of the fetus better than the category methods, as only about 30% of the patients in the pathological category suffer from acidosis, while the majority of acidotic babies were in the suspect category, which is considered lower risk. By predicting the direct indicator of acidosis, umbilical cord pH, this work demonstrates a methodology, which uses fetal heart rate and uterine activity, to identify acidosis. This paper introduces a forecasting model based on deep learning to predict heart rate and uterine contractions, integrated with the classification algorithm, resulting in a robust tool for predictive fetal monitoring. The hybrid algorithm resulted in a model capable of providing future conditions of the fetus, which obstetricians can use for diagnosis and planning interventions. The ensemble classification algorithm had a test accuracy of 85% (*n* = 24) in predicting fetal acidosis on the features extracted from the cardiotocography data. When integrated with the classification model, the results from the prediction model (long short-term memory network) can effectively identify fetal acidosis 2 or 4 min in the future.

## 1. Introduction

Approximately 25 out of 1000 infants are affected by fetal asphyxia associated with metabolic acidosis [1]. Acidosis is the process of increasing acidic concentration in the blood and tissues. The mother’s placenta delivers oxygen and nutrients and removes waste products, especially CO_2_, from the fetus. This process is susceptible to changes based on maternal blood gas concentrations, uterine blood supply, placental transfer, and gas transport to the fetus. The above processes can lead to acidosis and significant fetal morbidity and mortality. In most such pregnancies, mild oxygen deprivation to the fetus occurs with no brain harm or cerebral damage. However, hypoxia can be moderate to severe in approximately 3 to 4 of the 1000 infants, with a few organ system complications and possible neonatal encephalopathy [1]. The reference range for pH values of the fetuses was obtained based on a few studies with blood specimens taken after delivery. It is important to note that low pH does not necessarily indicate a severe condition in the fetus in all situations [2,3]. This paper aims to develop an integrated model that identifies the patterns in FHR and UC and accurately predicts fetal acidosis using an LSTM and an ensemble algorithm.

The objective of monitoring techniques is to reduce the occurrence of mild asphyxia and to prevent moderate and severe asphyxia. In 1903, researchers pointed out that the evaluation of fetal heart rate variation gives us a reliable means of estimating a child’s well-being [4]. A general rule is that the infant’s life is at risk when the heart rate falls below 100 or exceeds 160 [5]. Over the years, various techniques were developed to observe the fetal state. Among these, auscultation is the oldest technique that involves listening to the fetus’s heartbeat using a stethoscope. Ultrasound was introduced in 1956 and is still commonly employed to interpret heartbeats by using sound waves. Though this technique is very successful, it is not feasible, as an experienced clinician must stay with the patient. Fetal scalp blood sampling is an internal monitoring technique that involves introducing an endoscope after dilating the cervix. The device is firmly pressed to the fetus’s scalp, and an incision is made to collect a drop of blood to measure the pH. One of the most accurate monitoring methods is recording and extracting the fetal heart rate with less noise.

Electronic fetal monitoring (EFM) came into existence in the 1970s; electronic equipment is used to track fetal heart rate (FHR) and uterine contraction (UC) continuously during labor. They are together known as cardiotocography (CTG). When interpreted by an obstetrician, these data give a strong indication of fetal health [6]. Computer-aided analysis of CTG data provides a consistent evaluation and can also identify parameters that are difficult to capture by the human eye.

The obstetricians visually analyzed the FHR and UC readings to identify metabolic acidosis and hypoxic injury [7]. In a few cases, this can lead to misdiagnosis due to varying interpretations and is highly dependent on the clinician’s experience [8,9,10,11,12,13]. It was reported that almost 50% of the deaths occurring during labor are due to improper diagnosis [14]. Therefore, it has been challenging to interpret CTG data as not all abnormalities result in acidosis [15,16].

The clinical decision support systems were developed as a solution for this problem to provide further insights into the fetus’s condition by identifying specific features. The National Institute of Child Health and Human Development provides the guidelines for parts of FHR and uterine contraction patterns from the CTG data. Feature extraction involves gathering specific parameters/patterns from signal/time-series data that can be easier to analyze than the entire signal sample. Automated computerized analysis of the CTG recordings decreases the subjective nature of the fetal state based on visual interpretation.

Artificial neural networks (ANN), with their capability of learning and generalizing, were most prominently used for fetal state assessment [17]. Adding fuzzy logic to a current clinical expert system capable of assessing 5 min segments of FHR signals was developed [18]. A classifier based on fuzzy inference systems of the FHR signals was developed to predict intrauterine growth retardation and type I diabetes [19]. This model relied on gestational age and quantitative description of the fetal heart rate data in time and frequency domain FHR analysis for classification. Artificial neural networks with three layers and clustering using fuzzy logic were compared for over sixteen thousand FHR signals in a database with thirty-nine parameters [20]. Fetal state assessment based on FHR data analysis was performed using an ANN, combined with the inference system using fuzzy logic, developed for predicting fetal state/category based on fetal heart rate signals analysis. Epsilon-insensitive learning method based on statistical learning theory was used to obtain high prediction accuracy [21]. A support vector machine (SVM) algorithm was applied to predict the intrauterine growth inhibition risk of the fetus and assess the impact of input feature selection on prediction accuracy [22]. The support vector machine algorithm, combined with the wavelet transformation of input features, helped achieve a higher prediction accuracy of acidemia risk [23]. An effective fetal ECG data extraction technique was modeled using Clifford wavelets [24]. SVM combined with empirical mode decomposition was developed to achieve high compliance with heart rate data prediction with an expert clinical interpretation [25]. The thesis presents a new method for extracting features and evaluating fetal acidemia risk.

Dawes/Redman criteria algorithm was developed in 1982 for CTG analysis to predict whether the fetal state would be expected or pathological [26]. This led to the development of a system for intrapartum fetal monitoring, combining CTG with ST-analysis of the electrocardiogram (ECG), named STAN S31 by Neoventa Medical [27]. It generates alarms for hypoxic conditions related to muscle contractions and lack of oxygen. Sport is another clinically implemented system for computerized FHR analysis. Despite the extensive research, few fetal assessment systems were implemented for real-time monitoring.

Deep learning is a recent advancement in computational intelligence, using multiple layers of neural networks [28]. In the past few years, researchers have used this methodology to develop insights into complex medical diagnostic problems, such as computed tomography [29], glaucoma detection [30], mammography [31], breast cancer detection [32], analysis of ECG signals [33], bone fracture detection [34], and diagnostic medicine [35]. It has also been previously applied to the classification of the fetal state as it can identify essential features without human guidance [36,37].

Most of the current research within fetal monitoring using deep learning focuses on classification. Still, this model of long short-term memory networks has shown significant results in forecasting data [38]. This paper integrates the modeling approaches of using computational intelligence techniques for classification and deep learning algorithms for forecasting, thereby providing the capability of predicting the future fetal condition during labor.

**Figure 1:**
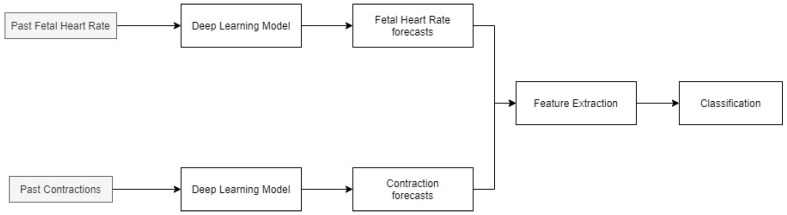
Methodology for predicting the future fetal condition.

The methodology of the paper is shown in Figure 1. FHR and UC data for the patients are forecasted using a deep learning model (LSTM). Feature extraction is then performed on the entire time series and newly predicted data. These features are then classified using an ensemble algorithm, consisting of a random forest and support vector machine to obtain the future fetal state. The identified variables can improve the diagnosis and fetal-monitoring process by providing additional information to the obstetricians in advance for them to plan and implement necessary interventions.

## 2. Materials and Methods

### 2.1. Support Vector Machine

Support vector machine (SVM) is one of the most commonly used machine learning algorithms for classification, which works by mapping inputs to the outputs of the training data using hyperplanes, thereby forming a generalized model. The kernel methods function maps the training data to the feature space. SVM uses a flexible representation of the class boundaries to solve classification problems. The aim is to develop a classifier that works well even with one unseen example.

**Figure 2:**
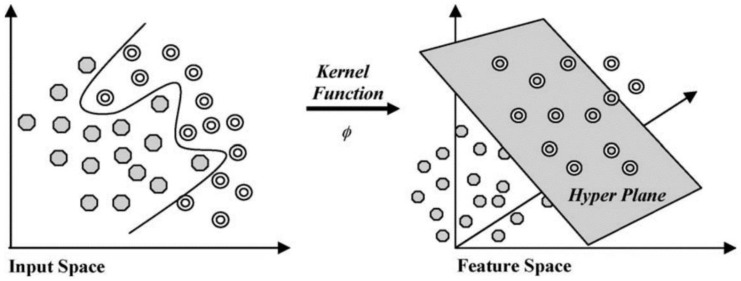
Input space to feature space conversion in SVM using kernel functions [40].

The hyperplane that maximizes the margin, or maximum separation between the classes, is selected, as represented in Figure 2. If it is inseparable, the margin boundary values and kernel methods are varied to identify the optimum parameters to separate the feature space. Different kernel functions commonly used are described in Table 1, where ‘x’ is the data value in the feature space, and ‘x_j_’ is the value in the transformed feature space [39]. The ‘γ’ parameter can be interpreted as inverse of the radius of influence, which represents the extent of influence of a single training sample.

### 2.2. Random Forest

Classification and regression tree (CART) is a repetitive partitioning supervised learning algorithm that makes no assumptions about the data distribution. Random forest involves building an ensemble of CART (classification and regression trees) developed from a randomized variant of the tree induction algorithm. Decision trees are perfect for random forest as they have lower bias and higher variance.

**Figure 3:**
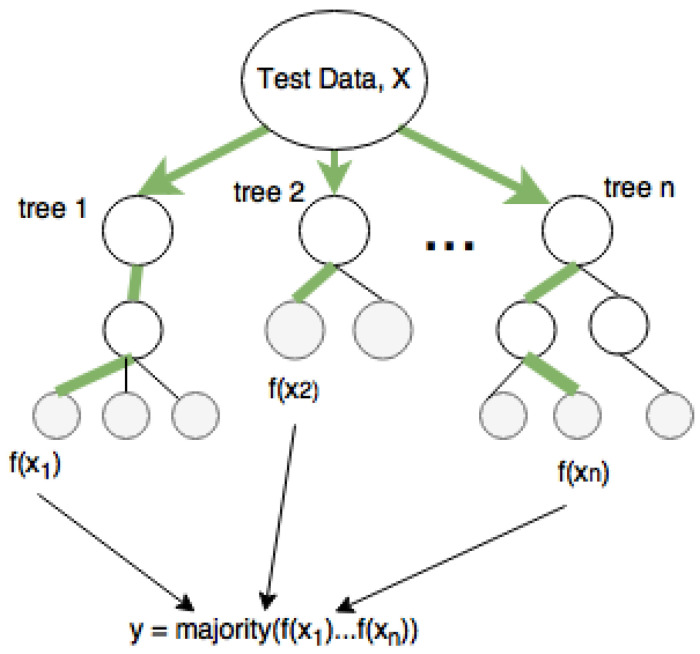
Classification of data with random forest.

In machine learning, random forests have been mainly applied to classification tasks due to their fast training and predictions, generalization ability, and scalability. A decision tree, as shown in Figure 3, can handle multiple classes due to its probabilistic output. The grey nodes are the leaf nodes that give the output variable, and the mean and majority of all trees are the outputs for regression and classification, respectively. Classification and regression tree (CART) is a repetitive partitioning supervised learning algorithm, which makes no assumptions about the data distribution. Random forest involves building an ensemble of CART (classification and regression trees), developed from a randomized variant of the tree induction algorithm. Decision trees are perfect for random forest as they have lower bias and higher variance.

### 2.3. K-Means Clustering

Clustering is an unsupervised learning algorithm that partitions the observations (training data) into different clusters based on certain similarities. The process begins with the random selection of centroids for the clusters and assigning data points to the clusters based on Euclidian distance from the different centers. A new centroid is then evaluated by calculating each cluster’s mean of the data points. The process is repeated once again until the newly evaluated center does not change.

### 2.4. Long Short-Term Memory Network

Most of the computational intelligence algorithms are inspired by nature, including neural network, which is based on the operation of the human brain. In simple terms, NN is a function that maps the independent variables to the dependent variable. Deep learning models are essentially neural networks with an increased number of hidden layers. A recurrent neural network (RNN) is a deep learning model that can identify time-dependent information and is used for forecasting problems.

**Figure 4:**
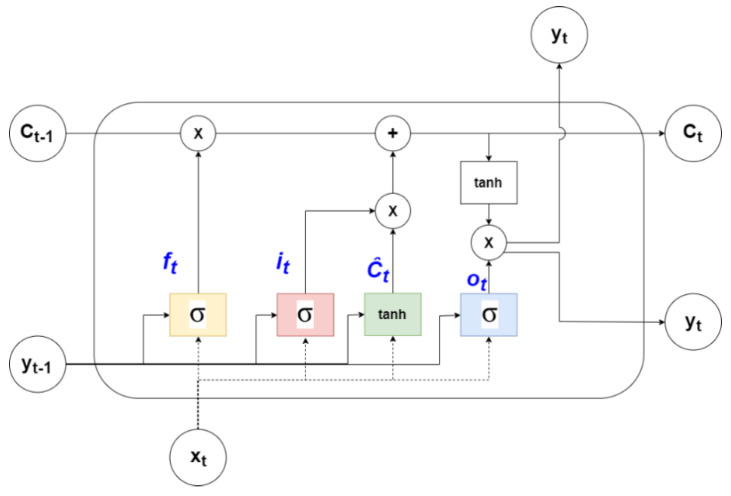
LSTM cell [41].

Long short-term memory network (LSTM) is a modified version of RNN with gates capable of retaining long-term information and is shown in Figure 3. The structure of an LSTM cell is shown in Figure 4. Each cell consists of 3 gates (forget, input, and output) regulating memory.

For a time-series forecasting problem, the model uses the input data from previous time steps (t) to predict the future (t + 1). The input vector for such a model can be represented as X = {x_1_, …x_n_} and output vector as Y = {y_1_, …, y_n_}. The ‘forget’ gate decides the information can be removed from the memory based on the output of the previous step and current input. It is formulated, as shown in Equation (1), where ‘U’ and ‘W’ are matrices containing the weights of inputs and recurrent connections, respectively.

f_t_ = σ(x_t_ U^f^ + y_t−1_ W^f^) (1)

The ‘input’ gate decides what information needs to be stored and has ‘sigmod’ and ‘tanh’ layers. It is represented by Equations (2) and (3).

I_t_ = σ(x_t_ U^i^ + y_t−1_ W^i^) (2)

Ĉ_t_ = tanh (x_t_ U^g^ + y_t−1_ W^g^) (3)

The memory of the LSTM cell is known as ‘cell state’ (C_t_), which is then updated based on the output from ‘forget’ and ‘input’ gates, given by Equation (4).

C_t_ = f_t_ C_t−1_ + i_t_ Ĉ_t_ (4)

Finally, the ‘output’ layer, consisting of a sigmoid layer and a tanh layer, generates the forecast (y_t_) for the time step ‘t’ and is formulated, as shown in Equations (5) and (6).

o_t_ = σ(x_t_ U^o^ + y_t−1_ W^o^) (5)

y_t_ = tanh(C_t_) × o_t_ (6)

### 2.5. Data

**Figure 5:**
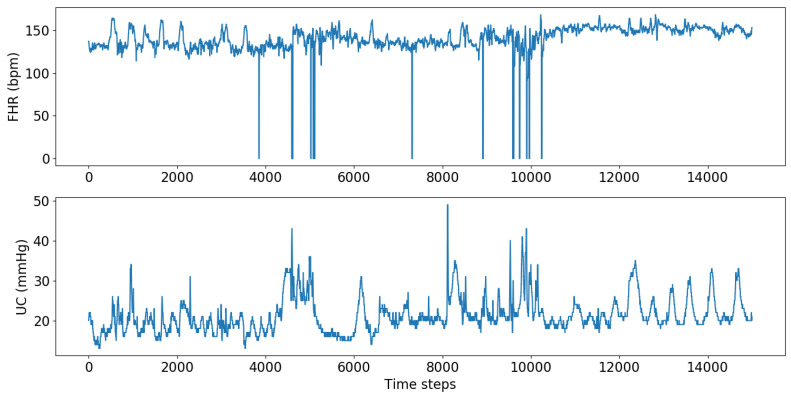
CTG data.

CTG data consist of four readings of FHR and UC collected every second during labor. The Phelps County Regional Medical Center provided over 8000 patients’ CTG data with their corresponding pH values. Forty-seven patients were diagnosed with acidosis; therefore, the dataset size was limited to 94 with even distribution of acidosis and non-acidosis cases to maintain the balance. An example of the raw data are shown in Figure 5. The cut-off point for differentiating acidosis was chosen as 7.2; all the values below 7.2 are considered acidotic, and the values above are non-acidotic.

## 3. Feature Extraction

The list of features extracted from the CTG data are FHR baseline, accelerations, decelerations, uterine contractions, variable decelerations, severe decelerations, late decelerations, prolonged decelerations, prolonged accelerations, light decelerations, width of the histogram, minimum, maximum values of the histogram, number of peaks of the histogram, mean, median, mode, and variability. These features are based on the current maternal and fetal medicine practices of the International Federation of Gynecology and Obstetrics (FIGO) [42].

**Figure 6:**
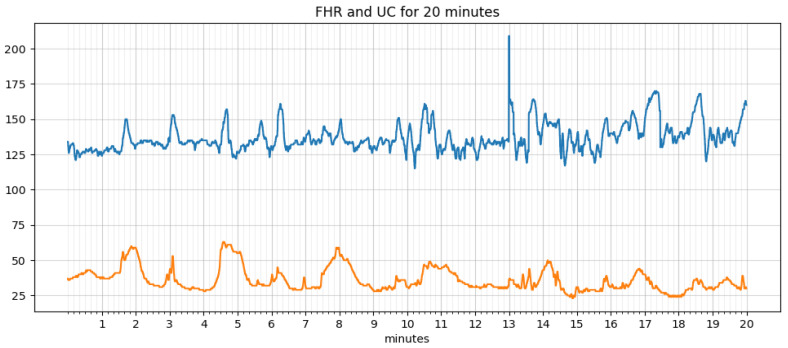
Raw data.

**Figure 7:**
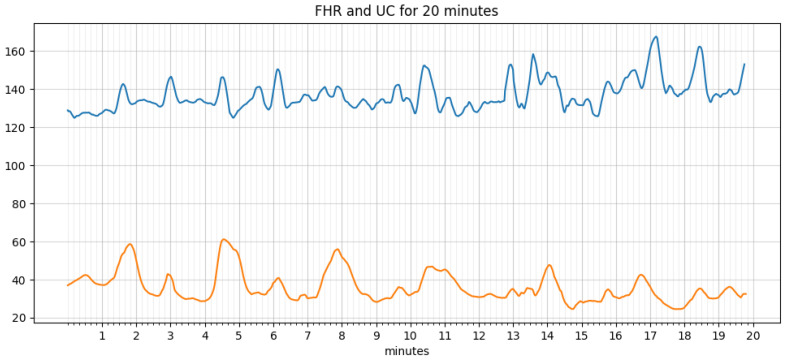
Data after smoothing.

An algorithm for extracting features, such as baseline, acceleration, deceleration, early deceleration, late deceleration, and variability, was written and implemented in python. An iterative approach estimates the baseline as defined by the FIGO guidelines. The signal loss and noise in the FHR and UC data are taken care of by smoothing using the ‘pandas’ library in python. The data before and after processing are shown in Figure 6 and Figure 7, respectively. It is simple and optimal for reducing random noise while retaining a sharp step response.

**Figure 8:**
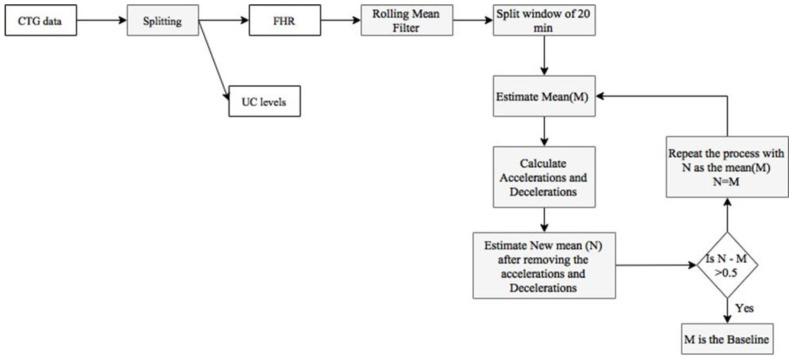
Methodology for determining the baseline.

The feature extraction process begins with identifying the baseline, as described in Figure 8. The original baseline (M) was calculated as the mean of the FHR data. Then, a new mean (N) was evaluated after removing accelerations and decelerations and compared to the original baseline to check the deviation. If the deviation exceeds 0.5, the process was repeated with the new baseline (N) as the baseline (M).

After evaluating the baseline heart rate, accelerations and decelerations were identified. Acceleration has a peak of at least 15 beats/min above baseline and a duration of at least 15 s but less than 2 min. The flowchart for estimating accelerations, baseline, and decelerations is shown in Figure 8. A deceleration has a fall of at least 15 beats/min below the baseline and a duration of at least 15 s but less than 2 min. A deceleration between 2 min and 5 min was defined as prolonged deceleration. A deceleration lasting more than 5 min is called a severe deceleration. If the deceleration starts after the peak and before the endpoint of the contraction, lasting more than 15 s, it is considered late deceleration. Peaks of over 10 points in UC level readings lasting 20–240 s were identified as contractions.

A histogram is plotted for the fetal heart rate data from which mean, median, and mode are calculated. Minimum and maximum values of the histogram are identified. The width of the histogram is evaluated as the difference between the minimum and maximum values. Finally, the classification parameter for the problem is acidosis. A pH value less than 7.2 is defined as acidotic, and a pH value of 7.2 or greater is non-acidotic. 

**Figure 9:**
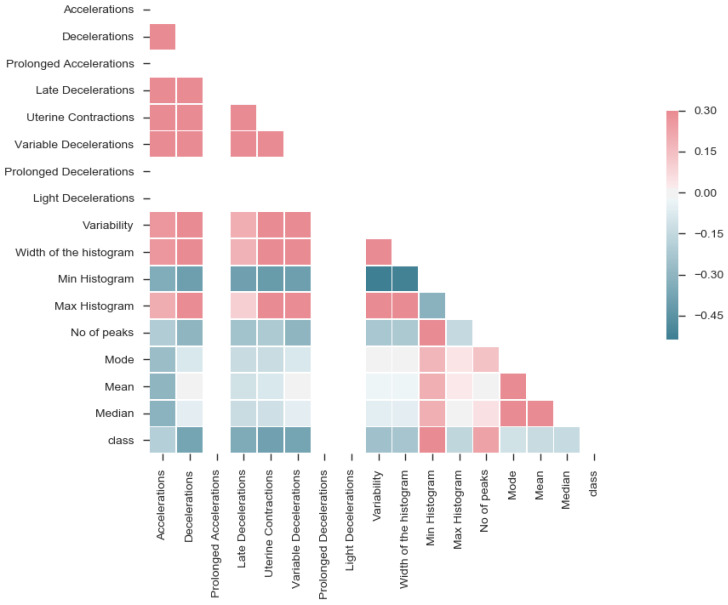
Correlation matrix.

After extracting features, the correlation was performed to understand the complexity of the data. The correlation matrix for the data is shown in Figure 9. This visualization helped identify features, such as prolonged accelerations, prolonged decelerations and light decelerations, having no association with any of the features. Further analysis showed that these features appear in two samples only, thereby not influencing the classification. So, the features mentioned above can be removed.

## 4. Results

### 4.1. Classification

Support vector machine (SVM), random forest (RF), and neural network (NN) were used to classify the CTG recording based on the features. Hyperparameter tuning was performed using a grid search for all the algorithms. Similarly, the parameters tuned for NN are hidden layer size, learning rate, solver, and activation function. Accuracy, sensitivity, and specificity are the performance measures used to compare the algorithms. The 5-fold cross-validation was used to avoid overfitting. 

The results are summarized in Table 2. We can observe that the support vector machine and neural network have higher accuracy, sensitivity, and specificity than random forest. The overall lower accuracy can be attributed to less training data.

### 4.2. Ensemble Approach

**Figure 10:**
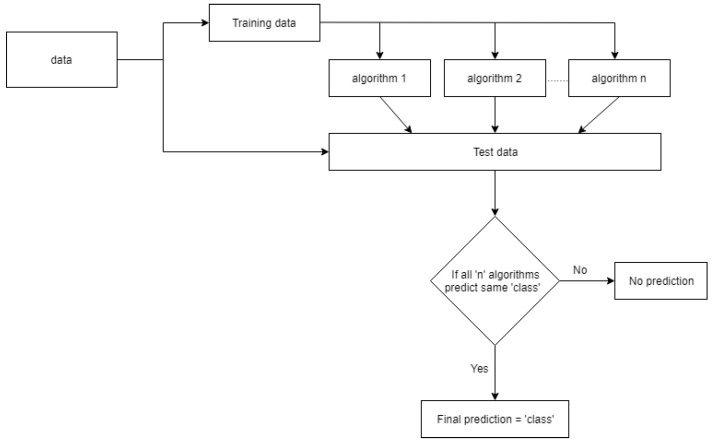
Ensemble classification algorithm methodology.

An ensemble algorithm is a technique to develop a better algorithm from a few weaker ones. The ensemble algorithm methodology is represented in Figure 10. The data were initially separated into training and testing data, and all the chosen algorithms were trained on the same data. The trained algorithms are evaluated individually on the test data. If all the algorithms predict the same class, the ensemble algorithm outputs the corresponding class, but if they do not predict the same category, the algorithm does not generate an output for that observation.

Neural network, support vector machine, random forest from previous experiments, and K-means clustering were chosen for the analysis. Ten combinations of the ensemble models were tested, including five sets of two algorithms, four combinations of three algorithms, and, finally, one combination of all four algorithms.

The results can be seen in Table 3. The combination of the three algorithms neural network, support vector, and clustering (NN/Clu/SVM) has performed the best with the highest accuracy. NN/Clu/SVM classified 14 out of 24 samples. NN/Clu predicted the largest number of instances (22), with the lowest accuracy of 80.95%.

**Figure 11:**
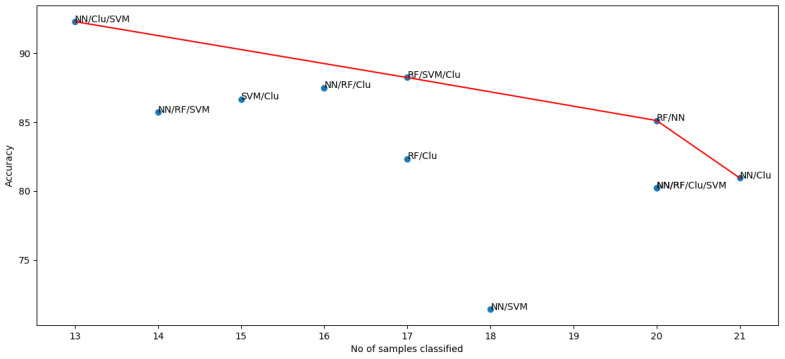
Accuracy and number of samples classified for the different ensemble algorithms.

The two desired qualities are the number of samples classified and the performance. Figure 11 shows the 2-dimensional Pareto front for these objectives. It can be observed that NN/RF/Clu/SVM, NN/Clu/SVM, RF/SVM, and RF/NN are the non-dominated solutions. We chose RF/NN as the optimal combination with a reasonable trade-off between both objectives.

### 4.3. Deep Learning

This section discusses the development of a deep learning model for forecasting and its integration with the classification model to predict the future state of the fetus. As discussed in earlier sections, CTG data consist of FHR and UC. Therefore, two different LSTM models must be developed for each CTG data sample. The algorithms were implemented in Python using the Keras library. Hyperparameters, such as the number of hidden layers, number of neurons in each layer, batch size, loss function, and optimizers, were tested using grid search with a range of values. The data processing involved standardization and smoothing with moving averages. The ‘SGD’ was the optimizer used, and ‘means squared error’ was the loss function used.

The best architectures for predicting FHR and UC are shown in Table 4 and Table 5. The input layer represents ‘lookback,’ which is the number of time steps the model looks back to make the forecast. Table 4 and Table 5 show the lookback values to be 1500 and 2000, respectively. The LSTM layer indicates the number of LSTM cells in that corresponding layer. The dropout layer with a value of ‘0.1’, meaning 10% of neurons from the previous layer, is neglected. Finally, a single forecast is generated by the model from the dense layer.

**Figure 12:**
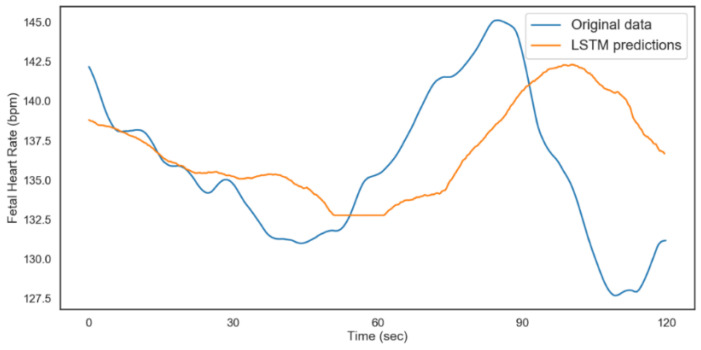
LSTM fetal heart rate predictions (acidosis).

**Figure 13:**
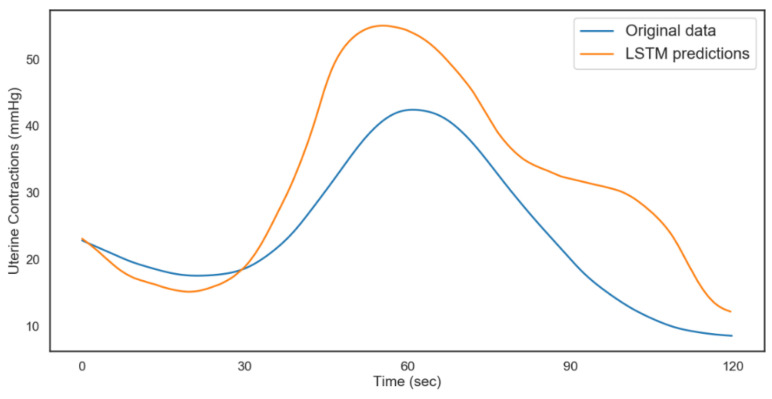
LSTM uterine contractions predictions (acidosis).

The model was validated on 480 time steps, which translates to 2 min (120 s). A total of 80% of the rest of the data was used for training the model and 20% for testing. The results from both models are shown in Figure 12 and Figure 13. Table 6 shows the performance metrics for the testing and validation of the algorithms. The better performance of the model for FHR during validation could be attributed to the distribution of data and the regularization technique (dropout) during the training. From the results, we can conclude that with ideal hyperparameters, LSTM can be a robust model for understanding and predicting CTG data.

**Figure 14:**
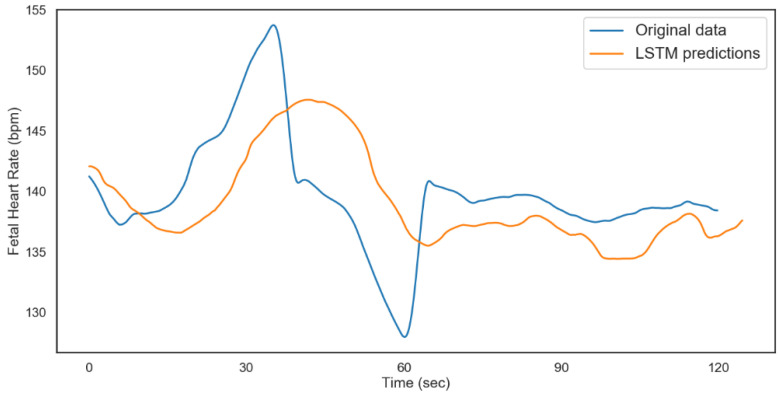
LSTM fetal heart rate predictions (non-acidosis).

**Figure 15:**
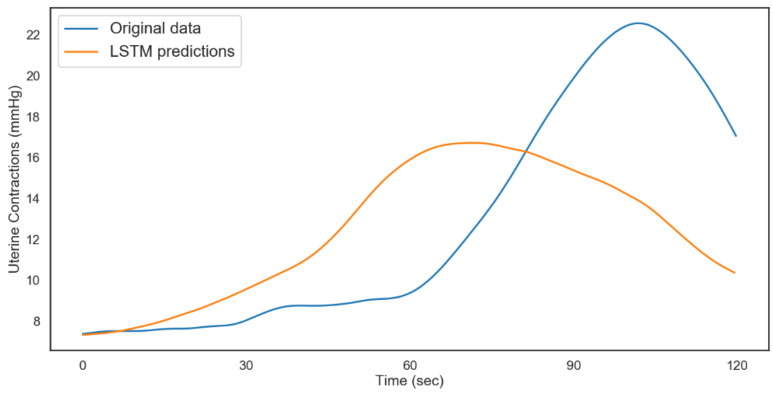
LSTM uterine contractions predictions (non-acidosis).

The modeling process was repeated for the non-acidosis data sample. Table 7 and Table 8, and Figure 14 and Figure 15, show the tabulated architectures and result visualizations, respectively. Similar to the results from acidosis data, we can observe that the forecasts for FHR and UC were close to the actual data. The corresponding errors for testing and validation are summarized in Table 9.

In the final experiment, we perform feature extraction on forecasts from the LSTM models for 2 and 4 min into the future. The NN and SVM ensemble algorithms were used to classify those features. This generated final output would be the state predicted by the model. Four readings are recorded every second, so the forecasts for 2 min and 4 min represent the ‘480’ and ‘960’ time steps, respectively. The results are summarized in Table 10. For the non-acidosis data sample, both NN and SVM predicted the class accurately for both 2 and 4 min. Therefore, the resultant ensemble output is ‘0’ or ‘non-acidosis.’ However, for acidosis, NN predicted the class accurately for both time periods, but SVM failed to classify the state for 4 min. Therefore, the ensemble output for 4 min in the acidosis scenario is ‘Unsure.’ As discussed in earlier sections, the model can be improved by providing more data for training the classification and forecasting algorithms.

## 5. Discussion

In this research, we presented an integrated model consisting of classification and forecasting models for evaluating the future state of the fetus. LSTM generates the forecasts for FHR and UC data, and an ensemble classification predicts the state based on the extracted features. As far as we know, this model is the first of its kind with this capability.

An ensemble model aims to develop a better model from weaker algorithms. Our results validate this observation, since the ensemble algorithm with NN and SVM showed significantly better results than the individual models. Most of the results in existing literature are based on UCI fetal state classification datasets. An accuracy of 96% was achieved on this data using a support vector machine [22]. The primary reason for the lower accuracy of the ensemble model discussed in this paper is the dataset size being limited to 94 samples. The model performance can be improved by training on more observations. In our experiments, we have observed an increase in the accuracy of oversampling the data with Gaussian noise. However, given the context of the problem, we believe obtaining additional data would be the right solution. Other methodologies for classification, such as image analysis and sequence classification with LSTM using deep learning, can be tested to see if the performance increases with limited data.

**Figure 16:**
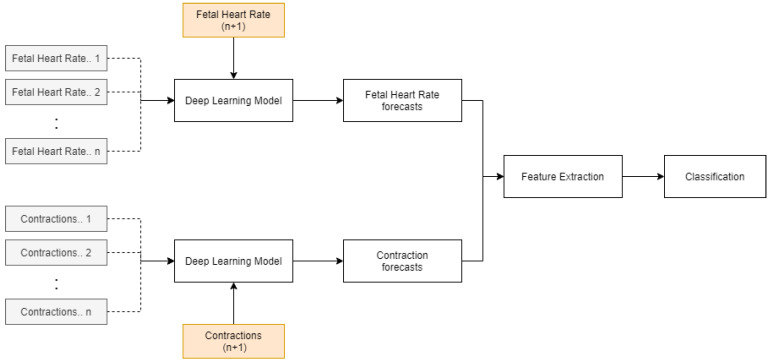
LSTM with multi-time-series training.

The paper’s objective was to develop an integrated model that identifies the patterns in FHR and UC and forecasts the corresponding values using an LSTM, which are then classified using the ensemble algorithm. The discussed methodology can provide obstetricians with the capability of understanding the future fetal condition and current state. The possible outcomes of the model for the fetal state are ‘acidosis’, ‘non-acidosis’, and ‘unsure’, which are easy to interpret. It can help doctors to make informed decisions regarding interventions based on these predictions. A drawback of the current approach is the development of a new LSTM architecture and optimization of hyperparameters for FHR and CTG of every training sample. This can be avoided by implementing an LSTM model trained on multiple time series data, as shown in Figure 16. The initial training time will be significantly higher. However, this will be a single generalized model, unlike the current approach.

Further extensive training and validation on a larger ECG database can result in a robust model, which can be implemented in a real-time fetal-monitoring decision support system.

## Figures and Tables

**Table 1:** Formulation of kernel functions.

Kernel	Function (x, x_j_)
Linear	x^T^x_j_
Polynomial	(γ x^T^ x_j_ + r)^d^, γ > 0
Gaussian RBF	exp(−||x − x_j_||^2^/2γ^2^)

**Table 2:** Performance metrics.

Performance Metrics	SVM	RF	NN
Accuracy	72.22	66.67	69.85
Sensitivity	66.66	50.00	58.33
Specificity	85.71	83.33	83.33
Precision	67.77	60.89	69.67

**Table 3:** Performance of different ensemble algorithm combinations.

Combination	No of Samples Classified (24)	Accuracy
**NN/SVM**	18	71.42
**NN/Clu**	21	80.95
**RF/NN**	20	85.12
**RF/Clu**	22	81.81
**SVM/Clu**	17	82.35
**NN/RF/SVM**	15	86.67
**NN/RF/Clu**	14	85.71
**RF/SVM/Clu**	16	87.50
**NN/Clu/SVM**	17	88.24
**NN/RF/Clu/SVM**	13	92.30

**Table 4:** LSTM architecture for fetal heart rate (acidosis).

Layers	Output
Input Layer	(None, 1, 1500)
LSTM Layer	(None, 10)
Dropout Layer	(None, 10)
Dense Layer	(None, 2)
Dense Layer	(None, 1)
Forecasts	1

**Table 5:** LSTM architecture for uterine contractions (acidosis).

Layers	Output
Input Layer	(None, 1, 2000)
LSTM Layer	(None, 10)
Dropout Layer	(None, 10)
Dense Layer	(None, 1)
Forecasts	1

**Table 6:** Performance measures (acidosis).

Measure	FHR	UC
Testing RMSE	7.6314	5.5155
Testing MAE	6.2494	3.9329
Validation RMSE	5.3828	6.4757
Validation MAE	4.0908	5.2563

**Table 7:** LSTM architecture for fetal heart rate (non-acidosis).

Layers	Output
Input Layer	(None, 1, 1000)
LSTM Layer	(None, 10)
Dense Layer	(None, 1)
Forecasts	1

**Table 8:** LSTM architecture for uterine contractions (non-acidosis).

Layers	Output
Input Layer	(None, 1, 800)
LSTM Layer	(None, 10)
Dropout Layer	(None, 10)
Dense Layer	(None, 1)
Forecasts	1

**Table 9:** Performance measures (non-acidosis).

Measure	FHR	UC
Testing RMSE	4.7568	1.1126
Testing MAE	3.7265	0.8337
Validation RMSE	4.7704	4.1983
Validation MAE	3.9593	3.2487

**Table 10:** Future fetal state classification.

Measure	Non-Acidosis (0)		Acidosis (1)	
	2 min (480 Time Steps)	4 min (960 Time Steps)	2 min (480 Time Steps)	4 min (960 Time Steps)
RF	0	0	1	1
NN	0	0	1	0
**Ensemble output**	**0**	**0**	**1**	**Unsure**

## Data Availability

The data are available upon reasonable request to the corresponding author.
